# The extra-embryonic area opaca plays a role in positioning the primitive streak of the early chick embryo

**DOI:** 10.1242/dev.200303

**Published:** 2022-06-20

**Authors:** Hyung Chul Lee, Cato Hastings, Claudio D. Stern

**Affiliations:** Department of Cell and Developmental Biology, University College London, Gower Street, London WC1E 6BT, UK

**Keywords:** Gastrulation, Embryo polarity, Embryonic axis, Symmetry breaking, Embryonic regulation, Regeneration

## Abstract

Classical studies have established that the marginal zone, a ring of extra-embryonic epiblast immediately surrounding the embryonic epiblast (area pellucida) of the chick embryo, is important in setting embryonic polarity by positioning the primitive streak, the site of gastrulation. The more external extra-embryonic region (area opaca) was thought to have only nutritive and support functions. Using experimental embryology approaches, this study reveals three separable functions for this outer region. First, juxtaposition of the area opaca directly onto the area pellucida induces a new marginal zone from the latter; this induced domain is entirely posterior in character. Second, ablation and grafting experiments using an isolated anterior half of the blastoderm and pieces of area opaca suggest that the area opaca can influence the polarity of the adjacent marginal zone. Finally, we show that the loss of the ability of such isolated anterior half-embryos to regulate (re-establish polarity spontaneously) at the early primitive streak stage can be rescued by replacing the area opaca by one from a younger stage. These results uncover new roles of chick extra-embryonic tissues in early development.

## Abstract (precis)

**Summary:** Two adjacent extra-embryonic tissues, the area opaca and the marginal zone, interact to influence the polarity of the early chick embryo.

## INTRODUCTION

Before the start of gastrulation (formation of the primitive streak), the chick embryo is disc-shaped and comprises three concentric regions: an inner (central) disc called area pellucida (which includes all cells destined to contribute to the embryo proper), an outermost ring called the area opaca and an intermediate narrow ring called the marginal zone (Lee et al., 2020; Eyal-Giladi and Kochav, 1976; Kochav et al., 1980). A considerable body of classical work has implicated the latter, and especially its posterior portion (‘posterior marginal zone’), in determining the site of primitive streak formation within the adjacent area pellucida (Bachvarova et al., 1998; Khaner and Eyal-Giladi, 1986, 1989; Khaner et al., 1985; Shah et al., 1997; Skromne and Stern, 2001; Spratt and Haas, 1960; Torlopp et al., 2014; Bertocchini and Stern, 2012; Azar and Eyal-Giladi, 1979; Eyal-Giladi and Khaner, 1989). The posterior marginal zone is a strong signalling centre that expresses cVG1 (currently labelled GDF3 in the chicken genome), misexpression of which in the anterior marginal zone is sufficient to initiate formation of an ectopic primitive streak (Seleiro et al., 1996; Shah et al., 1997; Skromne and Stern, 2001). The rest of the marginal zone displays a posteriorly-decreasing gradient of expression of BMP4 and its targets such as GATA2; BMP4 can act as an inhibitor of primitive streak formation (Bertocchini and Stern, 2012; Sheng and Stern, 1999). In the posterior area pellucida, two early targets of cVG1 signalling from the marginal zone are essential for initiating primitive streak formation at this site: cVG1 itself (Skromne and Stern, 2002) and another TGFβ superfamily member, NODAL (Bertocchini and Stern, 2002), which, as shown in other vertebrates, may act together with VG1/GDF as a heterodimer to induce mesendodermal fate (Montague and Schier, 2017; Opazo et al., 2019).

In contrast, the more peripheral region, area opaca, is generally believed to play a less active role in regulating embryonic patterning, its major functions at this early stage being providing a source of nutrition and maintaining the centrifugal tension of more central regions through adhesion of its edge to the vitelline membrane (Bellairs et al., 1967; Downie, 1976; New, 1959). Indeed, the embryo can generate a primitive streak even when the area opaca is removed, which has been interpreted to imply that the area opaca has no role in embryonic polarity (Spratt and Haas, 1960; Khaner et al., 1985).

The present study uncovers three separable roles of the area opaca in the regulation of polarity of the early embryo. First, it can induce a marginal zone (without polarity) when placed directly adjacent to the area pellucida. Second, it can induce posterior character on adjacent marginal zone and/or area pellucida embryo fragments. Finally, it appears to be responsible for the loss of the ability of isolated embryo fragments to form a primitive streak after the time of appearance of the endogenous primitive streak (stage HH2) (Bertocchini et al., 2004; Spratt and Haas, 1960).

## RESULTS

### Molecular differences between anterior and posterior area opaca

Previous studies have revealed a few genes, the expression of which differs in anterior and posterior parts of the extra-embryonic area opaca of the chick embryo. Among these, *BMP4* and *GATA2* are more strongly expressed anteriorly (Bertocchini et al., 2004; Sheng and Stern, 1999; Streit et al., 1998; Torlopp et al., 2014), whereas *WNT8C* is expressed more strongly posteriorly (Hume and Dodd, 1993; Skromne and Stern, 2001). A recent study explored regional differences in different parts of the embryo more systematically using RNA-seq (Lee et al., 2020). To uncover the most significant molecular differences between the anterior and posterior ends of the area opaca, we ranked the relative expression levels of these two regions. Table 1 lists several genes showing the highest level of differential expression – these observations suggest that the area opaca is polarized along the anterior-posterior axis.

**Table 1.**
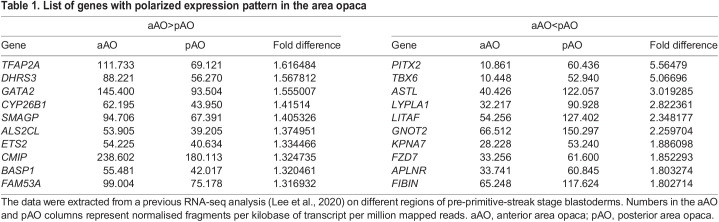
List of genes with polarized expression pattern in the area opaca

### The area opaca can induce functional properties of the marginal zone

A considerable body of work spanning several decades has revealed a crucial role for the marginal zone (a belt of cells intervening between the embryonic central area pellucida and the extra-embryonic outer area opaca) in regulating the polarity of the early blastoderm by positioning the site at which the primitive streak will form (Khaner and Eyal-Giladi, 1986, 1989; Khaner et al., 1985; Spratt and Haas, 1960; Bachvarova et al., 1998). The observations in the previous section that the area opaca is polarized raises the possibility that it may also influence the polarity of either the marginal zone or the area pellucida (directly or indirectly).

**Fig. 1.**
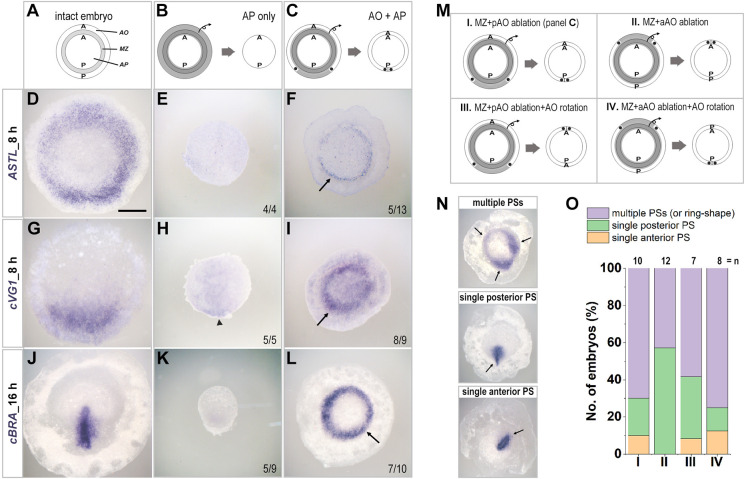
**Induction of a marginal zone by the area opaca.** (A,D,G,J) Expression of the marginal zone marker *ASTL* (D), of the posterior marginal zone marker *cVG1* (G) after 8 h culture, and of the primitive streak marker *cBRA* (J) after overnight culture, in normal embryos. (B,E,H,K) Expression of the same markers in embryos after ablation of the area opaca and the marginal zone. Some weak expression of *cVG1* is observed in the posterior area pellucida (arrowhead, H). Neither *ASTL* nor *cBRA* is expressed (or very low expression, in case of *cBRA*). (C,F,I,L) Expression of the markers in embryos after ablation of the marginal zone and grafting area opaca. In many cases, all markers (including the posterior markers *cVG1* and *cBRA*) show multiple or ring-shaped expression patterns (arrows). The proportion of embryos showing the morphology illustrated is indicated in each panel. (M-O) Effects of varying the orientation of the grafted regions or ablation of the area opaca. (M) Experimental design. (N) The resulting embryos could be assigned to three morphological classes. Arrows indicate primitive streak formation with *cBRA* expression. (O) Graph showing the incidence of each type of result. A, anterior; AO, area opaca; AP, area pellucida; MZ, marginal zone; P, posterior. Scale bar: 1 mm.

First, we explored whether the area opaca can induce marginal zone properties from area pellucida cells and, if so, whether the induced marginal zone is functionally polarized. To investigate this, we ablated the marginal zone. This frees a larger piece of area opaca than is necessary to surround the area pellucida (in the absence of the intervening marginal zone), so we grafted area opaca strips lacking the posterior or the anterior parts (∼60° arc). We assessed the results by examining the expression of the marginal zone marker *ASTL* and the posterior marginal zone marker *cVG1* after 8 h (Fig. 1). The isolated area pellucida did not express *ASTL* (0/4; Fig. 1E; Fig. S1B), confirming complete ablation of the marginal zone and suggesting that this region does not spontaneously regenerate. In contrast, a graft of area opaca (without the posterior region) induced a thin ring of *ASTL* expression in the adjacent area pellucida in some embryos (5/13 with expression) (*P*=0.0954, Boschloo's test) (Fig. 1F). Likewise, the isolated area opaca did not express *ASTL* (0/13; Fig. S1C), confirming that no marginal zone was included with the isolated area opaca. The induced expression of *ASTL* resembles that in the marginal zone of normal embryos (Fig. 1D). In the normal embryo, strong expression of *cVG1* is initially (stage X-XI) concentrated in the posterior marginal zone, later followed by expression in the neighbouring posterior area pellucida (Fig. 1A,G) and later in the primitive streak itself. When the area pellucida was cultured alone, *cVG1* expression was localised posteriorly, but very weakly expressed (Fig. 1B,H). In contrast, a graft of area opaca (lacking the posterior part) induced robust expression of *cVG1* in the area pellucida either as a ring or at multiple sites (8/9 with ring-like or multiple sites of expression, 1/9 in the posterior region only) (*P*=0.0007, Boschloo's test) (Fig. 1C,I).

To test whether expression of *cVG1* is followed by primitive streak and mesendoderm formation, we cultured the operated embryos overnight (16 h) and examined the expression of *cBRA* (*TBXT* or *BRACHYURY*). Unlike normal embryos, in which *cBRA* is expressed in the streak (Fig. 1J), the area pellucida alone showed no (or faint) expression – 5/9 had no expression (Fig. 1K) and the remaining 4/9 had weak expression localised posteriorly. When the area pellucida was surrounded by a strip of area opaca lacking the posterior part (Fig. 1C), most embryos (7/10) showed multiple sites or ring expression of *cBRA*, 2/7 had a single posterior streak and 1/7 had a single streak arising anteriorly (*P*=0.0007, Boschloo's test) (Fig. 1L). When the area pellucida was surrounded by a strip of area opaca lacking the anterior part (also a 60° arc), some embryos (3/7) also showed multiple foci or ring expression of *cBRA*, but a greater proportion (4/7) had a single posterior site of *cBRA* expression (Fig. 1M-O, conditions I and II).

As a further test of the ability of the area opaca to influence polarity of the area pellucida, we combined these juxtaposition experiments with 180° rotation of the area opaca relative to the area pellucida (Fig. 1M-O, conditions III and IV). In both cases (rotation of area opaca lacking either its anterior or its posterior portion), the majority of grafts generated a ring or multiple sites of *cBRA* expression (7/12 and 6/8 respectively), and the remainder generated a single primitive streak positioned either posteriorly or anteriorly in the area pellucida (Fig. 1M-O, conditions III and IV). The four different conditions (Fig. 1M-O) show no significant differences (*P*=0.2889, Fisher's exact test); the majority showed ring-shaped expression, emphasizing the strong inducing ability of the area opaca regardless of its orientation.

Together, these results show that the area opaca can induce a functional marginal zone in the area pellucida. However, the ring-shaped, rather than polarized, expression of *cVG1* and *cBRA* in the majority of embryos receiving an area opaca graft suggests that induction of the marginal zone by the area opaca can be separated from establishment of polarity in the marginal zone.

### The area opaca can bias the polarity of an isolated anterior half-embryo

In the above experiments, the area pellucida itself has already experienced the influence of more peripheral tissues (marginal zone and perhaps area opaca) by the time the experiment starts. To test whether the area opaca can influence polarity *de novo*, we turned to the anterior half of the blastoderm cultured by itself. As previously described, such a fragment will initiate primitive streak formation from either the left or the right posterior edge of the area pellucida with equal frequency (Bertocchini et al., 2004; Spratt and Haas, 1960; Torlopp et al., 2014). Therefore this provides a ‘sensitized’ system to assess subtle influences of the area opaca on polarity.

**Fig. 2.**
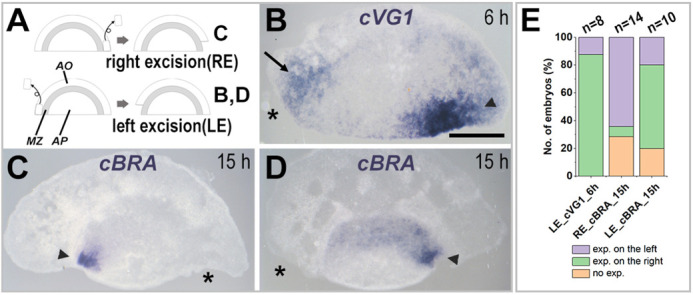
**Excision of area opaca from an isolated anterior half-embryo biases *cVG1* expression and primitive streak formation.** (A-D) Experimental design (A). Isolated anterior half-embryos are cultured after excision of either the right (RE; for C) or left (LE; for B,D) edge of the area opaca. Embryos were processed for expression of *cVG1* after 6 h (B) or *cBRA* (primitive streak) after overnight culture (C,D). After 6 h, *cVG1* is expressed more strongly at the contralateral side to the excision (arrowhead, B), but weak *cVG1* expression is also observed in the marginal zone lying immediately anterior to the excision site of area opaca (arrow, B). After overnight culture, a single primitive streak forms from the contralateral edge of the area pellucida (opposite the excision site; arrowhead, C,D). (E) Summary graph showing the number of embryos with different expression (exp.) patterns. Asterisks indicate site of excision. AO, area opaca; AP, area pellucida; MZ, marginal zone. Scale bar: 1 mm.

First, we ablated a small piece of posterior lateral area opaca from one side of the anterior half, the polarity of which was then assessed by expression of *cVG1* (after 6 h) or *cBRA* (after overnight culture). Expression of *cVG1* was observed, now more frequently in the marginal zone at the opposite side to the ablation (Fig. 2A,B,E). The marginal zone slightly anterior to the excision site (but not that immediately adjacent) showed weak expression of *cVG1* (Fig. 2B). After overnight culture, *cBRA* expression was also biased to the opposite side to the excision (*P*=0.0148, Fisher's exact test) (Fig. 2A,C-E). These results suggest that the area opaca influences the polarity of an isolated anterior half-blastoderm.

### Primitive streak-inducing ability of the area opaca

**Fig. 3.**
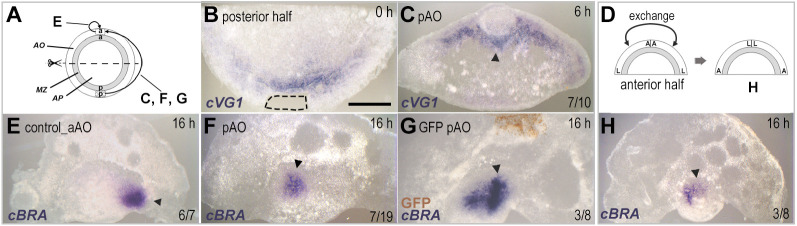
**A graft of posterior area opaca onto an isolated anterior half-embryo induces *cVG1* expression and primitive streak formation.** (A) Experimental design for B,C,E-G. (B) The posterior piece of area opaca (not expressing *cVG1*) is used as the donor for grafting (dotted line). (C) After 6 h, *cVG1* is induced in the marginal zone adjacent to the graft (arrowhead). (D,H) When the area opaca of the isolated anterior half-embryo is cut in half and the left and right fragments exchanged (to swap the anterior and lateral aspects of the area opaca, D), an ectopic primitive streak with *cBRA* is induced (arrowhead, H). (E,F) After overnight culture, an ectopic primitive streak (*cBRA* expression) is generated near the graft (arrowhead, F), whereas control grafts (anterior area opaca) have no effect – the primitive streak forms from one edge of the isolated anterior half-embryo as it does in the absence of a graft (arrowhead, E). (G) Using donor tissue taken from GFP-transgenic embryos reveals no cellular contribution of the graft to the induced ectopic primitive streak (arrowhead, G). The proportion of embryos showing each illustrated morphology is indicated in each panel. AO, area opaca; AP, area pellucida; MZ, marginal zone. Scale bar: 1 mm.

To test whether the polarity of the area opaca itself exerts an influence on the site of primitive streak formation of the isolated anterior half, a piece of anterior area opaca was replaced by its posterior counterpart (from the other half of the embryo) (Fig. 3A). The donor posterior half-embryo was examined for *cVG1* expression immediately after excision to ensure that the graft did not include the *cVG1*-expressing region of the marginal zone (Fig. 3B) (Shah et al., 1997). After short incubation (6 h), *cVG1* expression was observed in the marginal zone near the grafted donor tissue (7/10 with *cVG1* expression, 3/10 with no expression) (Fig. 3C). After overnight incubation, *cBRA* expression was observed near the grafted donor tissue in 7/19 embryos (37%), whereas the remaining 12 embryos (63%) had formed a streak from the left or right posterior edge, not near the donor tissue (Fig. 3F). Relative to control grafts (excision and replacement of the anterior area opaca from the same embryo), which resembled simple isolated anterior halves in that the majority (6/7; 86%) had one site of *cBRA* expression either on the left or the right posterior edge (the remaining embryo had *cBRA* expression anteriorly, near the site of the graft; Fig. 3E), the effect is relatively small (*P*=0.1699, Boschloo's test). To check for any contribution of the donor cells to the primitive streak, GFP-transgenic embryos (McGrew et al., 2008) were used as donors of posterior area opaca. Immunostaining with anti-GFP antibody revealed no contribution of donor cells to the marginal zone or the primitive streak after overnight incubation (3/8, Fig. 3G; 5/8 had formed a primitive streak from the posterior edge but not near the graft).

To test whether the inducing ability of the area opaca includes a gradient of anterior-posterior polarizing information, the orientation of the area opaca was reversed in the isolated anterior halves by cutting and recombination, and the formation of the primitive streak was investigated (Fig. 3D). In some embryos, formation of an ectopic primitive streak was observed where the lateral area opaca of the isolated anterior halves was placed, suggesting that the ability of the area opaca to induce posterior character is stronger laterally than anteriorly (3/8, Fig. 3H; 5/8 had a streak on either edge but not in the anterior). Together, these results are consistent with the idea that the posterior area opaca emits inducing signals that can polarize the embryo and position the site of primitive streak formation, even in the presence of the endogenous marginal zone, but only when the posterior part of the marginal zone is not present.

### The area opaca is responsible for the loss of the regulative ability of the primitive-streak-stage embryo

**Fig. 4.**
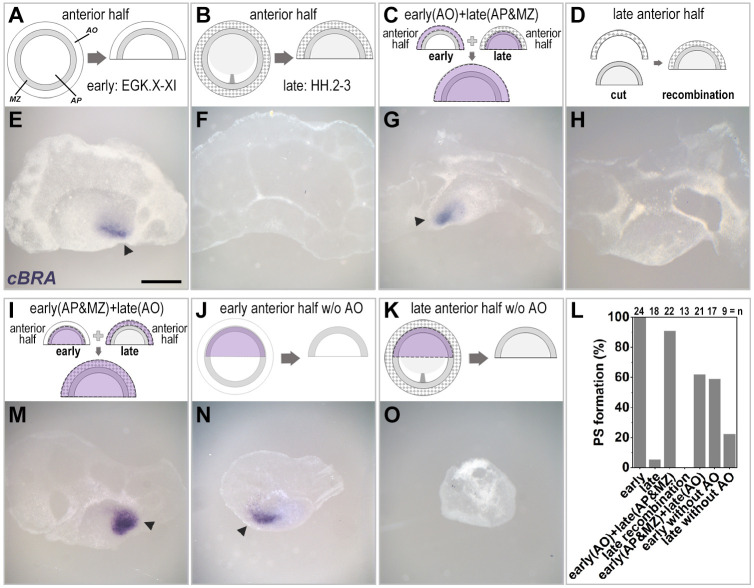
**The early area opaca can rescue the loss of regulative ability of primitive-streak-stage anterior half-embryos.** (A,E) The anterior half of pre-primitive-streak-stage (‘early’) embryos spontaneously generates a primitive streak expressing *cBRA* (arrowhead, E) from either the left or right posterior edge. (B,F) In contrast, the anterior half of primitive-streak-stage (‘late’) embryos can no longer generate a primitive streak. (C,G) Replacing the anterior area opaca of a late anterior half-embryo with the equivalent region from an early donor rescues the regulative ability of the late-stage embryo fragment, generating a primitive streak (arrowhead, G). (D,H) As a control for the effects of manipulation itself, excision and replacement of the anterior area opaca of a late anterior half-embryo does not generate a primitive streak. (I,J,L-N) Conversely, grafting late anterior area opaca onto a younger host (I,M) reduces the regulative ability of the latter to a level comparable with anterior half-embryos deprived of the area opaca at the same stage (J,L,N). (K,O) The majority of late anterior half-embryos deprived of the area opaca do not form an ectopic primitive streak (L). (L) Quantification of the results. AO, area opaca; AP, area pellucida; MZ, marginal zone. Scale bar: 1 mm.

The regulative ability of an isolated anterior half-blastoderm is lost as soon as the primitive streak starts to form (Spratt and Haas, 1960; Streit et al., 1998) (Fig. 4A,B,E,F,L), raising the question of whether the area opaca may be at least partly responsible. To test this, we grafted the anterior area opaca of a pre-primitive-streak-stage embryo (stages EGK X-XI; ‘early’) to the inner region (area pellucida and marginal zone) of the anterior half of a primitive-streak-stage host (stage HH 2-3; ‘late’), or vice versa (Fig. 4C,I). When early area opaca was placed adjacent to a late inner anterior half, most cases showed *cBRA* expression (20/22), indicating that the area opaca from a younger embryo can rescue the regulative ability of a later stage anterior half (Fig. 4C,G,L). To exclude the possibility that this result is due to the operation itself, we combined late area opaca with a late inner anterior half (area pellucida plus marginal zone) – no ectopic primitive streaks formed (0/13), confirming that the tissue recombination procedure is not the cause of ectopic streak formation in these experiments (Fig. 4D,H). In the converse experiment, when late anterior area opaca was placed next to a younger inner anterior half-blastoderm (area pellucida and marginal zone), the frequency of primitive streak formation was 62% (13/21) (Fig. 4I,L,M) – this represents a reduction in frequency relative to anterior halves of early embryos with their own area opaca, which generate a primitive streak in 100% of cases (24/24) (*P*=0.0005, Boschloo's test) (Fig. 4A,E,L). This decrease in frequency raises the possibility that the late area opaca exerts an inhibitory influence on primitive streak formation. To test this, we cultured the anterior half of early embryos in the absence of the area opaca (Fig. 4J). They exhibited a similar reduction in frequency of primitive streak formation (Fig. 4L,N), suggesting that the late area opaca does not gain inhibitory properties but rather loses its inducing, or polarity-promoting, functions. In contrast, when the anterior half of late embryos was cultured after removal of area opaca, most of the embryos (78%) did not make a primitive streak (Fig. 4K,L,O), which is comparable with the results with the inner anterior half of early embryos (59%; Fig. 4J,N) (*P*=0.0496, Boschloo's test). This result suggests that the regulative ability of the anterior half decreases over time, regardless of the presence of the area opaca. Together, our results uncover previously unreported roles for the extra-embryonic area opaca, including both the ability to induce a marginal zone and a polarising influence that can result in positioning the site of primitive streak formation.

## DISCUSSION

**Fig. 5.**
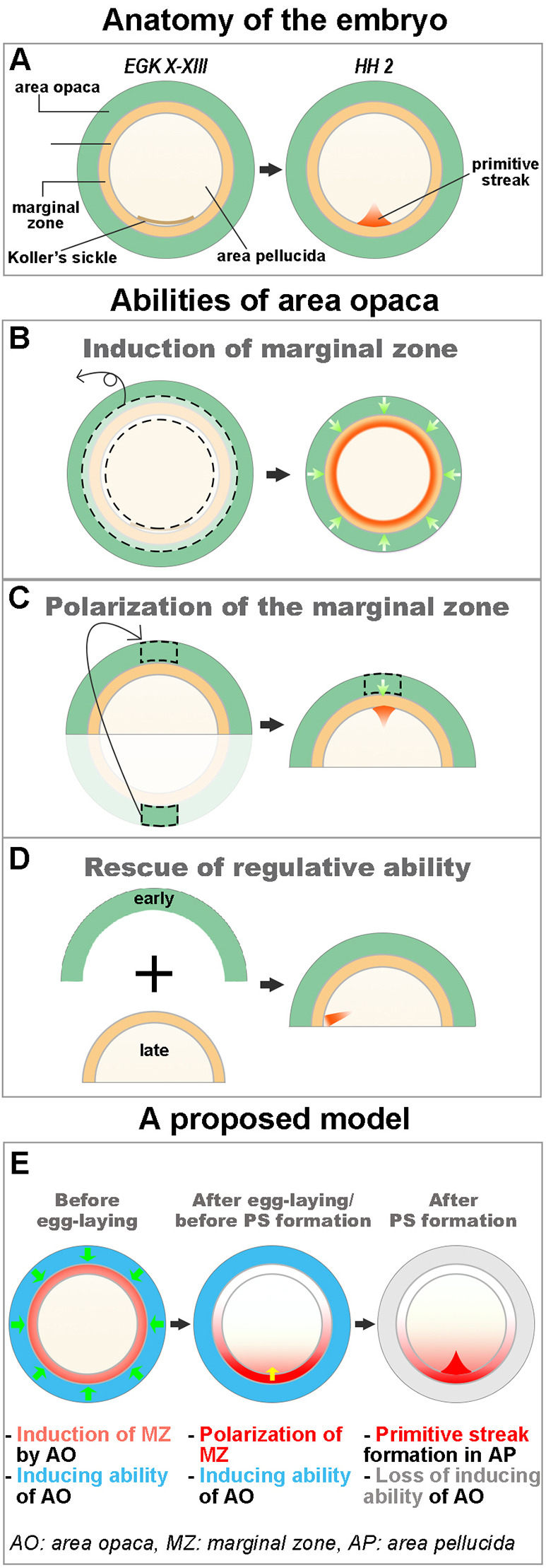
**Summary diagram showing the roles of the area opaca on embryo polarity and a proposed model.** (A) Anatomy of the early chick embryo at pre-primitive-streak (EGK X-XIII) and early-primitive-streak (HH 2) stages. Anterior at the top. (B-D) Abilities of the area opaca on embryonic polarity. (B) The area opaca can induce marginal zone when grafted to the area pellucida (in the absence of the marginal zone). (C) A piece of posterior area opaca can induce posterior identity (including primitive streak formation) when grafted onto an isolated anterior half-embryo. (D) Grafting the anterior area opaca of an early embryo (EGK X-XIII) to the inner regions (marginal zone plus area pellucida) of a later embryo (HH 2) can rescue the regulative ability of the latter, which would normally have been lost by this stage. (E) A proposed model of early developmental events involving the area opaca. This comprises three successive interactions: first (before egg laying), the area opaca induces the marginal zone property (which is posterior in character all the way around); subsequently (stage EGK X-XI), only one part of the marginal zone retains this posterior character, which becomes the dominant determinant of polarity; once the primitive streak forms, the inducing and polarising ability of the area opaca is lost.

The early chick embryo (before formation of the primitive streak) could be viewed as consisting largely of a single layer of epithelial cells that is continuous across its three concentric regions: the area pellucida at the centre (containing all prospective embryonic cells), the area opaca at the periphery, and an intermediate thin ring of cells, the marginal zone, lying between the previous two (Duval, 1889; Eyal-Giladi and Kochav, 1976; Pander, 1817; Pasteels, 1940; Stern, 2004; Vakaet, 1970; Kochav et al., 1980) (Fig. 5A). Beneath this single ectodermal layer are different tissues that largely do not contribute to the embryo: the single-cell-thick hypoblast and endoblast underlying the area pellucida (the former does carry germ cell precursors), and a thick multi-layered spongy arrangement of large yolky cells, called the germ wall, underlying the area opaca and marginal zone (Stern, 1990; Stern and Downs, 2012; Vakaet, 1970; Stern, 2004). Marking the boundary between area pellucida and marginal zone at the posterior edge of the former is a sharp ridge of cells, Koller's sickle (Bachvarova et al., 1998; Callebaut and Van Nueten, 1994; Izpisúa-Belmonte et al., 1993; Koller, 1882), which protrudes ventrally beneath the ectoderm and provides a bridge between this and the underlying hypoblast/endoblast. Although the marginal zone boundaries are not obvious morphologically in intact embryos other than at this posterior domain, brushing off the edges of the germ wall towards the area opaca reveals a continuous ring where the germ wall is not attached to the overlying ectoderm, corresponding to the region that had been called marginal zone in the classical literature (Eyal-Giladi and Kochav, 1976; Kochav et al., 1980). A recent study (Lee et al., 2020) uncovered the first molecular marker restricted to this entire region: ASTL, encoding an astacin-like metalloendopeptidase, confirming the existence of a distinct anatomical region that surrounds the entire area pellucida as proposed by previous observations.

Based on a number of classical studies it had been generally thought that the peripheral region of the embryo, the area opaca, plays a role in providing nutrition to the embryo and in maintaining its tension, but that it does not have an instructive role in regulating cell fate or embryonic polarity (Bellairs et al., 1967; Downie, 1976; Khaner et al., 1985; New, 1959; Spratt and Haas, 1960). Contrary to this conclusion, the present study uncovers three separable functions of the area opaca: induction of marginal zone properties, an influence on polarity of the marginal zone and determining the end of the period during which embryo fragments can ‘regulate’ (repolarise themselves and form a primitive streak from an isolated fragment lacking the primitive streak-forming region) (Fig. 5). We shall discuss these three properties in turn.

### Induction of marginal zone from the area pellucida

When placed directly adjacent to the area pellucida (ablation of the marginal zone; Fig. 1, Fig 5B), the area opaca can induce marginal zone properties in the latter. Interestingly, this induced marginal zone appears to be entirely posterior in character, all around the circumference, even if the grafted area opaca did not include its posterior part. This raises the possibility that posterior character may represent a ‘default’ condition of the marginal zone, and that this needs to be suppressed by other signals in the normal embryo to restrict posterior character to the appropriate position. This was quite unexpected, as the strong inducing functions of the posterior marginal zone and its expression of cVG1, controlled by the transcription factor PITX2 (Torlopp et al., 2014), appeared to reflect an active cell interaction defining this region.

### Polarization of the marginal zone

Ablation of a small piece of lateral area opaca from the edge of an isolated anterior half-embryo biases primitive streak formation to the opposite side (Fig. 2). In addition, grafting of a piece of posterior area opaca to the anterior region of an isolated anterior half-embryo can induce the formation of a primitive streak (Fig. 3, Fig. 5C). As the primitive streak formation in those experiments is preceded by *cVG1* expression in the marginal zone adjacent to the graft, the area opaca appears to influence embryo polarity indirectly through the marginal zone, rather than directly affecting the area pellucida. Also, given that the area opaca can polarize the embryo only in the absence of the posterior marginal zone, the polarizing influence of the area opaca is mainly supportive and much weaker than that of the marginal zone. This raises the possibility that, before polarization of the embryo (at intrauterine stages) in normal development, the area opaca may have an early role in specifying the marginal zone, which is later polarized. Unfortunately, embryos at these stages are difficult to obtain, to manipulate and to culture.

### Loss of regulative ability

Once the primitive streak forms in the embryo, isolated anterior fragments can no longer regulate: they no longer generate a primitive streak. One possible cause might be loss of competence in the anterior marginal zone in response to the area opaca. However, the anterior marginal zone of HH stage 3 embryos still can respond to cVG1 by generating a primitive streak after grafting a cVG1-expressing cell pellet (Bertocchini et al., 2004), indicating that the anterior marginal zone is competent even after primitive streak formation. Here, we show that the loss of regulative ability can be rescued by grafting anterior area opaca from a younger pre-primitive-streak-stage donor embryo adjacent to the marginal zone of the anterior half of an older primitive-streak-stage embryo (Fig. 5D). This suggests that changes in the signalling properties of the area opaca at later stages are a cause of the disappearance of the regulative ability of the embryo. Formally, these changes could reflect the gain of an inhibitory signal at later stages; however, the converse experiment of grafting the anterior area opaca from an older (HH2-3) donor to a younger host (anterior half of the area pellucida plus its marginal zone) does not cause a loss of regulative ability in the young anterior half. Taken together, these results suggest that the young area opaca emits positive signals required for the regulative ability of the area pellucida plus marginal zone (and loss of those signals account for the loss of such ability).

### Possible mechanisms: mechanical tension or secreted factors (Wnt, BMP)?

What mechanisms might account for the inducing ability of the area opaca and for the loss of support of regulation at later stages? One possibility is that the area opaca may signal to the marginal zone by emitting secreting molecules. WNT and BMP family members are possible candidates. WNT signalling has been shown to collaborate with cVG1 and to be necessary for primitive streak formation (Hume and Dodd, 1993; Skromne and Stern, 2001). One of its ligands, WNT8C, is expressed strongly in the area opaca and is also expressed as a gradient in the marginal zone (Lee et al., 2020; Skromne and Stern, 2001). Once the primitive streak forms at stage HH2-3, *WNT8C* disappears from the area opaca and is only limited to the outmost edge cells (one or two cells thick) of the area opaca (Lee et al., 2020). Thus, based on its early role on primitive streak formation and its disappearance from the area opaca after primitive streak formation, WNT signalling from the area opaca may be a signal to keep the regulative ability of the embryo. Like *WNT8C*, *BMP4* is strongly expressed in the area opaca and the marginal zone but with an opposite gradient (strongest anteriorly), and disappears from the area opaca at primitive-streak stages (Streit et al., 1998). Although BMP signalling acts as an inhibitor of primitive streak formation and *cVG1* expression, a recent study in the chick embryo suggested that the primitive streak is positioned by the balance between cVG1/NODAL and BMP signals, assessed locally by cells (Lee et al., 2022). Moreover, in mouse embryos, BMP signalling from extra-embryonic tissue induces WNT3 in the epiblast to amplify NODAL signalling and thus position the primitive streak (Ben-Haim et al., 2006).

A second possibility is that tension generated by the expanding edges of the area opaca (the ‘margin of overgrowth’; Bellairs et al., 1967; Downie, 1976; New, 1959) plays a role. However, embryo fragments lacking the area opaca can regulate (albeit at reduced frequency) (Spratt and Haas, 1960, 1961 and results in the present paper) and, moreover, Spratt's experiments were performed by growing the embryo in the absence of the vitelline membrane and placing directly (in some cases with its ventral surface downwards) on a semi-soft agar substrate to which it cannot adhere and which promotes very limited expansion. It is therefore more likely that the influence of the area opaca on the ability of the inner parts of the embryo to regulate is due to secreted signals.

### Speculations on the sequence of events during very early stages of development

The three separable activities of the area opaca uncovered in our experiments could reflect events that occur at very early stages of normal development. We have summarised this proposal in Fig. 5E. Before egg laying (prior to stage X), the area opaca may function to induce the marginal zone property (which is posterior in character all the way around). Shortly after laying (stage EGK X-XI), only one part of the marginal zone retains this posterior character, which then becomes the dominant determinant of polarity. Finally, as soon as the primitive streak forms (HH 2), the inducing and polarising abilities of the area opaca are lost, which partially accounts for the loss of the ability of non-posterior fragments of embryo to regulate.

## MATERIALS AND METHODS

### Embryo harvest and culture and whole-mount *in situ* hybridisation

Fertilised White Leghorn hen eggs were obtained from Henry Stewart Farm, UK, and incubated for 2-4 h or 13-14 h to obtain EGK stage X-XI (Eyal-Giladi and Kochav, 1976) or HH stage 2-3 (Hamburger and Hamilton, 1951) embryos, respectively, at 38°C. Transgenic chick embryos expressing cytoplasmic GFP were supplied by the avian transgenic facility at the Roslin Institute, Edinburgh, UK (McGrew et al., 2008). Embryos were harvested and manipulated (for tissue ablation and grafting experiments described in the text) in Pannett-Compton saline (Pannett and Compton, 1924), then cultured using a modification of the New culture method (New, 1955; Stern and Ireland, 1981) at 38°C for the desired period of time. They were then fixed in 4% paraformaldehyde in phosphate buffered saline (pH 7.4) at 4°C overnight before whole-mount *in situ* hybridisation as previously described (Stern, 1998; Streit and Stern, 2001). The probes used were: *cVG1* (Shah et al., 1997), *cBRA* (Kispert et al., 1995) and *ASTL* (ChEST817d16, from the chick EST collection, Source Bioscience) (Boardman et al., 2002; Lee et al., 2020). The stained embryos were observed under an Olympus SZH10 dissecting microscope and imaged with a QImaging Retiga 2000R camera.

### Embryo manipulation

Tissue ablation or excision was conducted in a 35 mm Petri dish filled with Pannett-Compton saline using a fine hypodermic needle or a bent insect pin. Tissue grafting was performed with the embryo on its vitelline membrane wrapped around a glass ring as described for New culture (New, 1955; Stern and Ireland, 1981). To facilitate adhesion of embryo fragments to each other, they were juxtaposed and any remaining liquid between them removed by a fine micro-needle pulled from a 50 µl capillary, attached to a mouth aspirator. If required, aspiration of liquid was repeated during the first 1-2 h of subsequent culture.

The anatomical criteria defining the boundaries between the marginal zone and its inner (area pellucida) and outer (area opaca) neighbouring regions have been carefully described mainly by the group of Eyal-Giladi and colleagues (Eyal-Giladi and Kochav, 1976; Azar and Eyal-Giladi, 1979; Kochav et al., 1980; Khaner et al., 1985) and these were later strongly confirmed by both fate mapping and molecular maps mainly from our own group (Stern, 1990; Bachvarova et al., 1998; Lee et al., 2020; Izpisua-Belmonte et al., 1993; Hatada and Stern, 1994). Briefly, the boundary between area opaca and the marginal zone is defined by the transition of adhesion of the deep layers of yolky cells (germ wall): in the outer area opaca, the deep cells are firmly attached to the epiblast all the way around, whereas in the marginal zone the germ wall overhangs as a ‘flap’ (‘germ wall margin’), without adhering to the overlying epiblast. The inner boundary of the marginal zone, separating it from the area pellucida, is defined by the presence of a ridge protruding ventrally from the epiblast, Koller's sickle (Bachvarova et al., 1998; Callebaut and Van Nueten, 1994; Izpisúa-Belmonte et al., 1993; Koller, 1882), present as an arc of ∼60°-90° centred at the posterior-most edge. To define the entire boundary in live embryos this is projected to continue around the blastodisc, but the boundary is also discernible by a change in structure of the epiblast that can be seen under transmitted light. From about stage XII, expression of ASTL marks this region clearly, coinciding with these anatomical landmarks (Stern, 2004; Lee et al., 2020).

These landmarks can only be seen clearly in some strains of domestic fowl (such as White Leghorn used in the present study) and not even in every embryo; we discarded embryos in which it was difficult to define the regions clearly and unambiguously. To confirm that the explants and excisions in our experiments were not contaminated by adjacent regions, we defined each region conservatively: in marginal zone ablation experiments, a thin ring of adjacent area opaca and area pellucida were included with the ablated tissue to ensure that no marginal zone remained. This was confirmed in some embryos using the marginal zone marker ASTL (Fig. 1; Fig. S1).

To keep track of the orientation of tissues before grafting, the posterior side was marked with carmine powder. After culturing the manipulated embryos for the desired time, the embryo was photographed both before and after *in situ* hybridisation to confirm the orientation of the embryo (as carmine colour is lost during the *in situ* procedure) and to observe morphology more clearly.

### Statistical tests

All statistical tests were performed in the R programming environment. We used Fisher's exact test for two-sided comparisons and Boschloo's test for one-sided ones.

## Supplementary Material

Supplementary information
