# Supplementary material for: The extra-embryonic area opaca plays a role in positioning the primitive streak of the early chick embryo
Source: Development. 2022 Jun 20;149(12):dev200303. doi: 10.1242/dev.200303 (PMC9270967; doi:10.1242/dev.200303)
Supplement: Supplementary information [file develop-149-200303-s1.pdf]

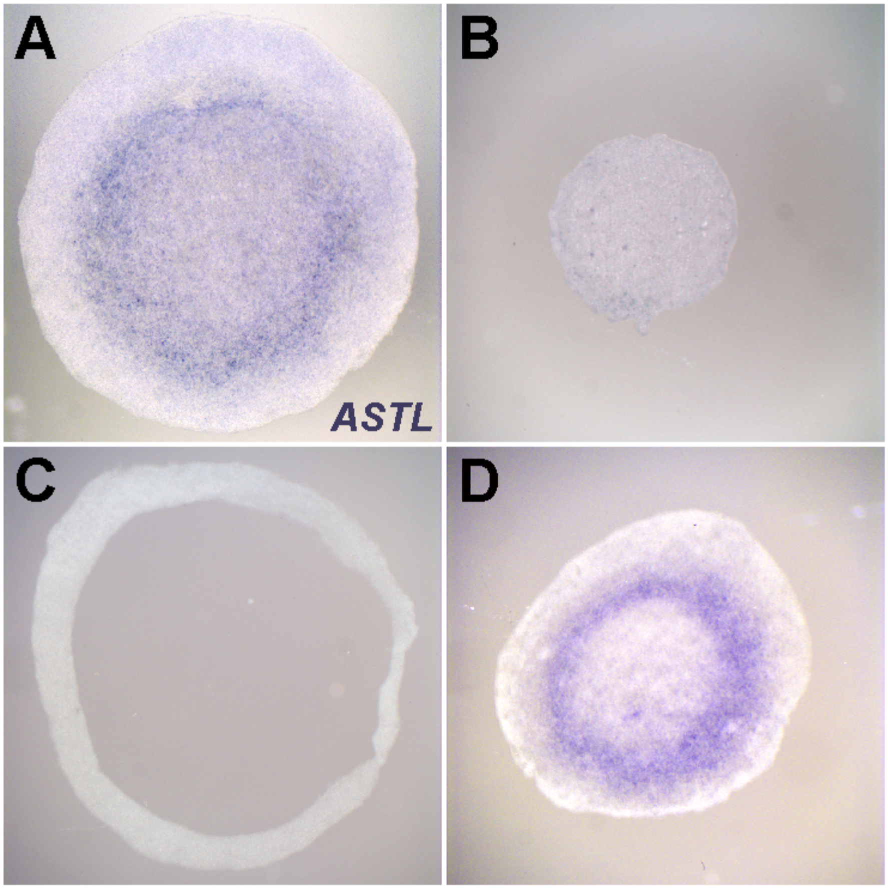

Fig. S1. No inclusion of *ASTL* expressing cells in isolated area pellucida or area opaca. An inner part of area pellcuida or an outer part of area opaca is excised from the pre-primitive-streak embryos and checked for the expression of the marginal zone marker *ASTL* after 8 h culture (as conduced in Fig. 1). (A) ASTL expression in a pre-primitive-streak embryo. (B and C) no expression of *ASTL* in the isolated area pellucida (B) or area opaca (C). (D) *ASTL* expression in the remaining tissue containing a bit of area opaca, marginal zone and area pellucida, after excision of the area opaca.

Development • Supplementary information
